# Downregulation of miR-200a Induces EMT Phenotypes and CSC-like Signatures through Targeting the β-catenin Pathway in Hepatic Oval Cells

**DOI:** 10.1371/journal.pone.0079409

**Published:** 2013-11-15

**Authors:** Jie Liu, Bai Ruan, Nan You, Qike Huang, Weihui Liu, Zheng Dang, Weihua Xu, Ti Zhou, Ru Ji, Yang Cao, Xia Li, Desheng Wang, Kaishan Tao, Kefeng Dou

**Affiliations:** 1 Department of Hepatobiliary Surgery, Xijing Hospital, Fourth Military Medical University, Xi’an, Shaanxi, People’s Republic of China; 2 Department of Hepatobiliary Surgery, Xinqiao Hospital, Third Military Medical University, Chongqing, People’s Republic of China; 3 PLA Center of General Surgery, General Hospital of Chengdu Army Region, Chengdu, People’s Republic of China; University of Alabama at Birmingham, United States of America

## Abstract

Hepatocellular carcinoma (HCC) can be derived from malignant transformed adult hepatic progenitor cells. However, the regulatory factors and molecular mechanisms underlying the process are not well defined. Our previous microRNA (miRNA) microarray analysis revealed a significant decrease of miR-200a level in F344 rat HCC side population (SP) fraction cells versus their normal counterparts. In the present study, we further investigated the effect of miR-200a on hepatic oval cell (HOC) phenotypes. We first confirmed downregulated miR-200a levels in rat hepatoma cells compared with WB-F344 cells. Next, by lentivirus-mediated loss-of-function studies, we showed that stable knockdown of miR-200a confers a mesenchymal phenotype to WB-F344 cells, including an elongated cell morphology, enhanced cell migration ability and expression of epithelial mesenchymal transition (EMT)-representative markers. Concomitantly, several cancer stem cell (CSC)-like traits appeared in these cells, which exhibit enhanced spheroid-forming capacity, express putative hepatic CSC markers and display superior resistance to chemotherapeutic drugs in vitro. Furthermore, bioinformatics analysis, luciferase assays and western blot analysis identified β-catenin (CTNNB1) as a direct and functional target of miR-200a. Knockdown of miR-200a partially activated Wnt/β-catenin signaling, and silencing of β-catenin functionally attenuated anti-miR-200a effects in vitro in WB-F344 cells. At length, in vivo xenograft assay demonstrated the acquisition of tumorigenicity of WB-F344 cells after miR-200a siliencing. Collectively, our findings indicate that miR-200a may function as an important regulatory factor in neoplastic transition of HOCs by targeting the β-catenin pathway.

## Introduction

Hepatocellular carcinoma (HCC) is the most common type of primary liver cancer, which accounts for the third most frequent cause of cancer-related death worldwide [1]. It is now well accepted that hepatocarcinogenesis is a complex, multi-step process associated with the accumulation of various genetic and epigenetic alterations [2]; however, the molecular pathogenesis of HCC remains mostly obscure. Elucidating and identifying novel molecules critically involved in the development of HCC could provide an alternative strategy for HCC prevention and therapy.

A growing body of evidence supports the hypothesis that cancers are initiated and maintained by a small subset of cells, termed cancer stem cells (CSCs) [3], [4]. Furthermore, CSCs might originate from normal stem/progenitor cells in certain pathological processes [5], [6]. In HCC, candidate hepatic CSCs have been isolated and identified by several research groups [7], [8]. Moreover, certain hepatic CSCs emerging during chronic liver injury share many common signaling pathways, including transforming growth factor beta (TGF-β) [9], β-catenin [10] and surface markers [11], with normal hepatic progenitor cells (HPCs) or hepatic oval cells (HOCs). In addition, there is also evidence demonstrating that dysregulated HPCs/HOCs possess tumor-initiating ability in vivo [12], [13]. These findings suggest that HPCs/HOCs might be involved in the genesis of hepatic CSCs. However, the specific molecular mechanism(s) remain(s) to be determined.

MicroRNAs (miRNAs or miRs) are a class of endogenous small noncoding RNAs (0–22 nt) that negatively regulate gene expression at the post-transcriptional level [14]. Recently, increasing studies have revealed that many miRNAs play crucial roles in tumorigenesis and cancer progression [15], [16]. More importantly, it has been demonstrated that several miRNAs participate in regulating self-renewal, differentiation and transformation in normal stem cells and CSCs [17], [18], [19], [20]. The miR-200 family is a group of evolutionarily conserved miRNAs, comprising five members (miR-200a, -200b, -200c, -141 and -429). In addition to extensive participation in inhibiting epithelial mesenchymal transition (EMT) in various cancer cells [21], the miR-200 family is also inversely associated with regulating CSC phenotypes of breast cancer [22], [23], pancreatic cancer [24] and ovarian cancer [25]. However, the function miR-200a exerts on hepatic stem cells and hepatic CSCs is rarely reported. Interestingly, using miRNA microarray and real-time quantitative polymerase chain reaction (qRT-PCR) analysis, our previous study showed that miR-200a was greatly downregulated in the F344 rat HCC side population (SP) fraction cells compared with their normal counterparts [26]. To this end, we hypothesized that miR-200a dysregulation might be implicated in the malignant transformation of hepatic stem cells.

Herein, we report the use of rat liver, oval-like progenitor cells (WB-F344) to investigate the function and regulation of miR-200a on their phenotypes. Using loss-of-function studies, we demonstrated for the first time that suppression of miR-200a is associated with CSC-like features and the EMT phenotype in WB-F344 cells in vitro, and is responsible for the acquisition of tumorigenicity in vivo. Furthermore, we identified β-catenin (CTNNB1) as the functional downstream target of miR-200a, and activation of the Wnt/β-catenin pathway is responsible, at least partially, for miR-200a-silencing-mediated biological functions in WB-F344 cells. These results provide new insight into miRNA function and open a new perspective for developing novel therapeutic strategies aimed at targeting EMT and hepatic CSCs.

## Materials and Methods

### Cell Lines and Cell Culture

The rat hepatic oval cell line WB-F344 (abbreviated WB cells) used in this study is structurally and phenotypically simple epithelial cells that were isolated from the liver of an adult male Fischer 344 rat [27]. Their morphological and biological properties have been previously characterized as mostly resembling the oval cells [28]. Furthermore, these cells could not form tumors when injected into nude mice [9].

The WB cell line, normal hepatic cell line BRL and hepatoma cell lines (H-4-II-E, CBRH-7919, RH-35) were obtained from the Cell Bank of the Chinese Academy of Sciences (Shanghai, China). Cells were routinely maintained in Dulbecco’s modified Eagle’s medium/Ham’s F12 medium (DMEM/F12, HyClone, Logan, UT) supplemented with 10% fetal bovine serum (FBS, Invitrogen, Carlsbad, CA), 100 U/mL penicillin and 100 U/mL streptomycin at 37°C in a humidified incubator containing 5% CO_2_. Only the cells in logarithmic growth phase were used throughout the research. Cells at low to passage 10 were used for the subsequent study.

### RNA Extraction and qRT-PCR Analysis

Total RNA, including miRNAs, was extracted from cells using TRIzol reagent (Invitrogen). Expression levels of rno-miR-200a were quantified using a miScript PCR System (QIAGEN, Hilden, Germany), including a miScript II RT Kit, miScript Primer Assays and miScript SYBR Green PCR Kit. Small nuclear RNA U6 was employed for internal normalization.

For mRNA analysis, complementary DNA (cDNA) was generated with oligo-dT primers using the Primescript RT reagent Kit (TaKaRa, Dalian, China). Amplification of the generated cDNA was performed using SYBR Premix EX Taq II (TaKaRa) on a Bio-Rad IQ^TM^5 Detection System (Bio-Rad, Hercules, CA). The rat housekeeping gene β-actin was used as an internal control to normalize mRNA expression levels of target genes. All of the above experiments were performed according to the manufacturer’s instructions.

The PCR conditions were 3 min at 95°C, followed by 40 cycles of 95°C for 15 s, 60°C for 30 s and 72°C for 60 s. All of the qRT-PCR reactions were run in triplicate, and data were analyzed according to the comparative Ct (2^−ΔΔCt^) method. The qPCR primers (listed in Table 1) were designed and synthesized by Sangon Biotech, Co., Ltd (Shanghai, China).

**Table 1:** Sequences of qRT-PCR primers used for mRNA analysis.

mRNA	Sequence	
ZEB2	Forward (5′-3′)	CCAACTCTGATGAACTGCTGAA
	Reverse (5′-3′)	CTTTTCTCTGCTCAAACCATTC
EpCAM	Forward (5′-3′)	CTGGCGTGGAACTCAGAACTTA
	Reverse (5′-3′)	GACACACACACACACACACACA
CD133	Forward (5′-3′)	CGAATGACTTCCCTCAAGATTT
	Reverse (5′-3′)	CCAGGATGACGCAGATAAGAAC
ABCG2	Forward (5′-3′)	TGGTTTGGACTCAAGCACAG
	Reverse (5′-3′)	CTGGTGAATGGAGAAGATGATG
CK19	Forward (5′-3′)	AACCACGAGGAGGAAATTAGTG
	Reverse (5′-3′)	TATCTGGATCTGCGTAGTGTGG
AFP	Forward (5′-3′)	AATCTGTTCCTCATTGGCTACA
	Reverse (5′-3′)	GCTCACCATCTTCCCTGTCA
ALB	Forward (5′-3′)	GACAAAGCAGCCTGCCTGAC
	Reverse (5′-3′)	TTCTGCGAACTCAGCATTGG
MYC	Forward (5′-3′)	GATGTGGTGTCTGTGGAAAAGA
	Reverse (5′-3′)	CTGTGTGGAGGTTTGCTGTG
β-actin	Forward (5′-3′)	GGAGATTACTGCCCTGGCTCCTA
	Reverse (5′-3′)	GACTCATCGTACTCCTGCTTGCTG

### Oligonucleotide Construction and Lentiviral Transduction

The oligonucleotide of mature miR-200a antagomir (5′-CCGGACATCGTTACCAGACAGTGTTATTTTTG-3′) was chemosynthesized, amplified and cloned into GV232-Puro Vectors by Genechem Co., Ltd. (Shanghai, China). The correct sequences and insertions were confirmed by DNA sequencing. WB cells were lentivirally transfected with either the GV232-Puro-anti-miR-200a recombined vector (WB-anti-miR-200a) or empty GV232-Puro vector (negative control, WB-miR-NC). Oligonucleotide transfection or lentivirus construction was performed using Lipofectamine 2000 reagent (Invitrogen) according to the manufacturer’s instructions. The transduced cells with a cell density of over 40% confluency were exposed to puromycin dihydrochloride (1 µg/mL, Sigma, St. Louis, MO) for resistance selection. When all of the cells in the non-transfected control culture were killed, puromycin-resistant cell clones were picked and passaged in medium containing a half concentration of puromycin (0.5 µg/mL) in the first round of selection. Lentivirus-mediated silencing of miR-200a was verified by qRT-PCR and western blot analysis.

### Protein Extraction and Western Blot Analysis

Cells were washed three times with ice-cold phosphate-buffered saline (PBS) and lysed in RIPA buffer (50 mM Tris (pH 7.4), 150 mM NaCl, 0.5% sodium deoxycholate, 1 mM EDTA, 1% Triton X-100) containing fresh protease and phosphatase inhibitor cocktails (Sigma). The lysate was then centrifuged at 12,000 rpm at 4°C for 25 min. Aliquots of the supernatant were denatured in boiling water for 5 min and quantified for the next analysis. Equal amounts of protein extracts (30 µg) were subjected to 10% sodium dodecyl sulfate-polyacrylamide (SDS-PAGE) gel electrophoresis and transferred to polyvinylidene difluoride (PVDF) membranes (Millipore, Billerica, MA) using a Bio-Rad apparatus. The membranes were incubated in 5% nonfat milk for 1 h at room temperature and washed with PBS, followed by incubation with primary antibodies against β-catenin (1∶800; Cell Signaling Technology), ZEB2, E-cadherin, N-cadherin, Vimentin, CyclinD1, c-Myc (1∶500; Santa Cruz Biotechnology), and β-actin (1∶1500; Zhongshan Goldenbridge, Ltd) overnight at 4°C. After rewarming and repeated washing, membranes were then incubated in the appropriate horseradish peroxidase (HRP)-conjugated secondary antibody (1∶5000, Abcam) at room temperature for 1 h. The blots were developed using enhanced chemiluminescence detection reagents (Pierce, Rockford, IL) and scanned with a Molecular Imager System (Bio-Rad).

### Cell Proliferation Assay

WB-miR-NC or WB-anti-miR-200a cells were seeded in a 24-well plate (Corning, Lowell, MA) at 1×10^4/^well in triplicate and maintained under standardized culture conditions. At selected time intervals, different cells were trypsinized into single-cell suspensions, and the cell number was calculated by a hemocytometer. The experiment was performed in triplicate.

### Cell Apoptosis Assay

The number of apoptotic cells was assessed by determining caspase activation and Annexin V-FITC/PI staining. For caspase activation detection, Caspase-Glo 3/7 assay kit (Promega, Madison, WI) was used according to the manufacturer’s instructions. Briefly, WB-miR-NC or WB-anti-miR-200a cells were plated at 1×10^4/^well into clear, opaque-wall 96-well plates (Corning) and incubated for 24 h. After the medium was removed, Caspase-Glo 3/7 reagent (100 µl) was added, gently mixed, and incubated at room temperature for 30 min. Five independent detections were performed, and the luminescence was determined using a Luminometer (Bio-Rad).

For flow cytometer detection of cell apoptosis, Annexin V-FITC/PI Apoptosis Detection Kit (Jingmei, Shanghai, China) was used. Briefly, the WB-miR-NC or WB-anti-miR-200a cells were collected, washed in cold PBS, incubated for 15 min with Annexin V-FITC and PI according to the manufacturer’s protocol, and then analyzed by a FACSCalibur flow cytometer and CellQuest software (BD Biosciences, San Jose, CA).

### Cell Spheroid-formation Assay

Single-cell suspensions of WB-miR-NC or WB-anti-miR-200a cells were plated at a density of 1×10^6^ cells per well in 6-well Ultra-Low Attachment Plates (Corning) and maintained in serum-free medium for 7 days. The number of spheroids was counted and statistically analyzed. Representative images were acquired under an inverted microscope (Olympus, Tokyo, Japan).

### Chemo-resistance Assay

WB-miR-NC or WB-anti-miR-200a cells were cultured in 6-well plates (Corning) at 1×10^5^ cells per well and were then treated with paclitaxel (10 ng/mL) or doxorubicin (30 ng/mL), respectively. The optimal doses of paclitaxel and doxorubicin were determined in our preliminary experiments (data not shown). After 48 h of exposure, cell apoptosis was tested by flow cytometer as described above. The tests were performed in triplicate.

### In vitro Migration Assay

The migration ability of the cells was assessed using uncoated Transwell Chambers (8 µm pore size; Millipore). Briefly, 600 µL of the cell culture medium supplemented with 10% FBS was added to the lower 24-well chamber as a chemo-attractant. WB-miR-NC or WB-anti-miR-200a cells were resuspended in serum-free medium, and then 200 µl of the single-cell suspension (2×10^4^ cells) was seeded onto the upper chamber of each transwell. After incubation for 24 h, the cells that did not migrate were removed from the upper surface of the membranes using a sterile cotton swab. The membranes were fixed with 4% formaldehyde and stained with 0.05% crystal violet. Finally, the cells attached to the lower surface of the membranes were counted at 200×magnification in five randomly selected areas per well. Each experiment was performed in triplicate.

### miR-200a Target Luciferase Reporter Assay

To validate predicted target genes, oligonucleotides (35 bp) containing wild-type or the mutated binding site for miR-200a from the rat β-catenin mRNA (CTNNB1) 3′-UTR were annealed and ligated into the *Eco*RI and *Pst*I sites of the pGL3-control-mcs2 reporter vector (named pGL3-CTNNB1-wt or pGL3-CTNNB1-mut). The oligonucleotide sequences were as follows: CTNNB1-wt (F: 5′-AATTCCTCGTAGTGTTAAGTTATAGTGAATCTGCA-3′ and R: 5′-GATTCACTATATAACTTAACACTACGAGG-3′) and CTNNB1-mut (F: 5′-AATTCCTCGTAGTGGGAAGTTATAGTGAATCTGCA-3′ and R: 5′-GATTCACTATATCCCTTAACACTACGAGG-3′).

WB cells were co-transfected with the above recombinant plasmids and 50 nM anti-miR-200a or anti-miR-control (Ambion, Austin, TX) using Lipofectamine 2000 (Invitrogen). A pRL-TK Renilla luciferase vector (Promega) was simultaneously co-transfected for normalization. After transfection for 48 h, luciferase activity was analyzed using a Dual-Luciferase Reporter Assay System (Promega) according to the manufacturer’s protocol. Three independent experiments were performed, and data were statistically analyzed.

### Immunofluorescence Staining

Cellular localization of β-catenin was determined by indirect immunofluorescence staining. Briefly, cells grown on coverslips were fixed in 4% fresh paraformaldehyde for 20 min and then permeabilized with 0.3% Triton X-100 in PBS for 10 min at room temperature. After the cells were blocked for 15 min with 10% normal goat serum, the coverslips were incubated overnight at 4°C with primary antibodies against β-catenin (1∶100, Cell Signaling Technology). After being extensively washed with PBS, the cells were incubated with species-specific Alexa Fluor 488-labeled secondary antibody (1∶200; Zhongshan Goldenbridge, Ltd) for 1 h at room temperature. Finally, the coverslips were washed, counterstained with 4′, 6′-diamidino-2-phenylindole (DAPI; 0.1 µg/mL, Sigma) for 5 min and mounted on glass slides. Immunofluorescence images were photographed under a fluorescence microscope (Olympus).

### Transient Transfection of siRNAs

For β-catenin inhibition studies, transient transfection of WB-anti-miR-200a cells with small-interfering RNA (siRNA) targeting CTNNB1 mRNA (siCTTNB1, Santa Cruz Biotechnology) or control siRNA (siControl, Santa Cruz Biotechnology) was performed. Briefly, 1×10^5^ cells were plated per well in 6-well plates in media containing 10% FBS to achieve 50% confluence, and then transfection of siCTNNB1 or siControl was performed using Oligofectamine (Invitrogen) to reach a final RNA concentration of 100 nM according to the manufacturer’s protocol. At 48 h post transfection, the cells were harvested for protein extraction and western blot analysis as previously described. Cell spheroid formation and migration were also assayed at 48 h post transfection. The effect of siCTTNB1 was confirmed by three independent transfection experiments, and representative results are shown.

### Top/Fop Luciferase Reporter Assay

TopFlash (wild-type) and FopFlash (mutant) vectors are a set of TCF-reporter plasmids that have been widely used for indicating β-catenin/TCF signaling activity. To assess β-catenin-mediated transcription activity in WB-anti-miR-200a cells, we employed the Top/Fop reporter gene system as previously described [29]. Briefly, one day before transfection, cells were plated in a 24-well plate and were then transiently transfected with 100 ng of β-catenin-responsive firefly luciferase reporter plasmid TopFlash (Millipore) or negative control FopFlash (Millipore), as well as 10 ng of internal control pRL-TK Renilla luciferase vector (Promega) using Lipofectamine 2000 (Invitrogen) following the manufacturer’s instructions. After transfection for 24 h, cells were harvested and analyzed for firefly and Renilla luciferase activity by the Dual-Luciferase Reporter Assay System (Promega). To further validate the function of siCTNNB1 on β-catenin-mediated transcription activity, WB-anti-miR-200a cells were co-transfected with TopFlash or FopFlash and siCTNNB1 using Lipofectamine 2000 (Invitrogen) in the same way. Cells were harvested at 48 h post transfection for luciferase activity detection. Each experiment was performed in triplicate, and the fold change in TopFlash activity compared with that in FopFlash activity is shown.

### In vivo Xenograft Tumorigenicity Assay

Athymic nude mice (BALB/C-nu/nu, 4–6 weeks old, male) were obtained from Animal Center of Chinese Academy of Science (Shanghai, China), housed under specific pathogen-free conditions and cared for in accordance with the guidelines of the laboratory animal centre of Fourth Military Medical University (Xi’an, China). All studies involving animals were approved by the Research Animal Care and Use Committee of Fourth Military Medical University. WB-miR-NC or WB-anti-miR-200a cells were mixed with Matrigel Basement Membrane Matrix (BD Biosciences) at ratio of 1∶1 and then subcutaneously implanted into left flanks (WB-miR-NC) or right flanks (WB-anti-miR-200a) of five nude mice at 2×10^6^ cells per injection, respectively. The mice were maintained under standardized conditions, monitored every 2 days after second week of inoculation and sacrificed 40 days post inoculation. Resultant subcutaneous tumors were pictured, collected and measured in weight. Tumor tissues were fixed in 4% formaldehyde, embedded in paraffin for hematoxylin and eosin (H&E) staining to analyze the histopathology under the microscope (Olympus).

### Statistical Analysis

Data are presented as the mean ± standard deviation (SD) from at least three independent experiments. Student’s *t*-test (two tailed) or Student–Newman–Keuls (SNK test, ANOVA) was employed to analyze differences using SPSS 17.0 software (Chicago, IL). A probability p-value less than 0.05 was considered statistically significant.

## Results

### Expression Levels of miR-200a and Validation for Stable miR-200a Knockdown in WB Cells

**Figure 1:**
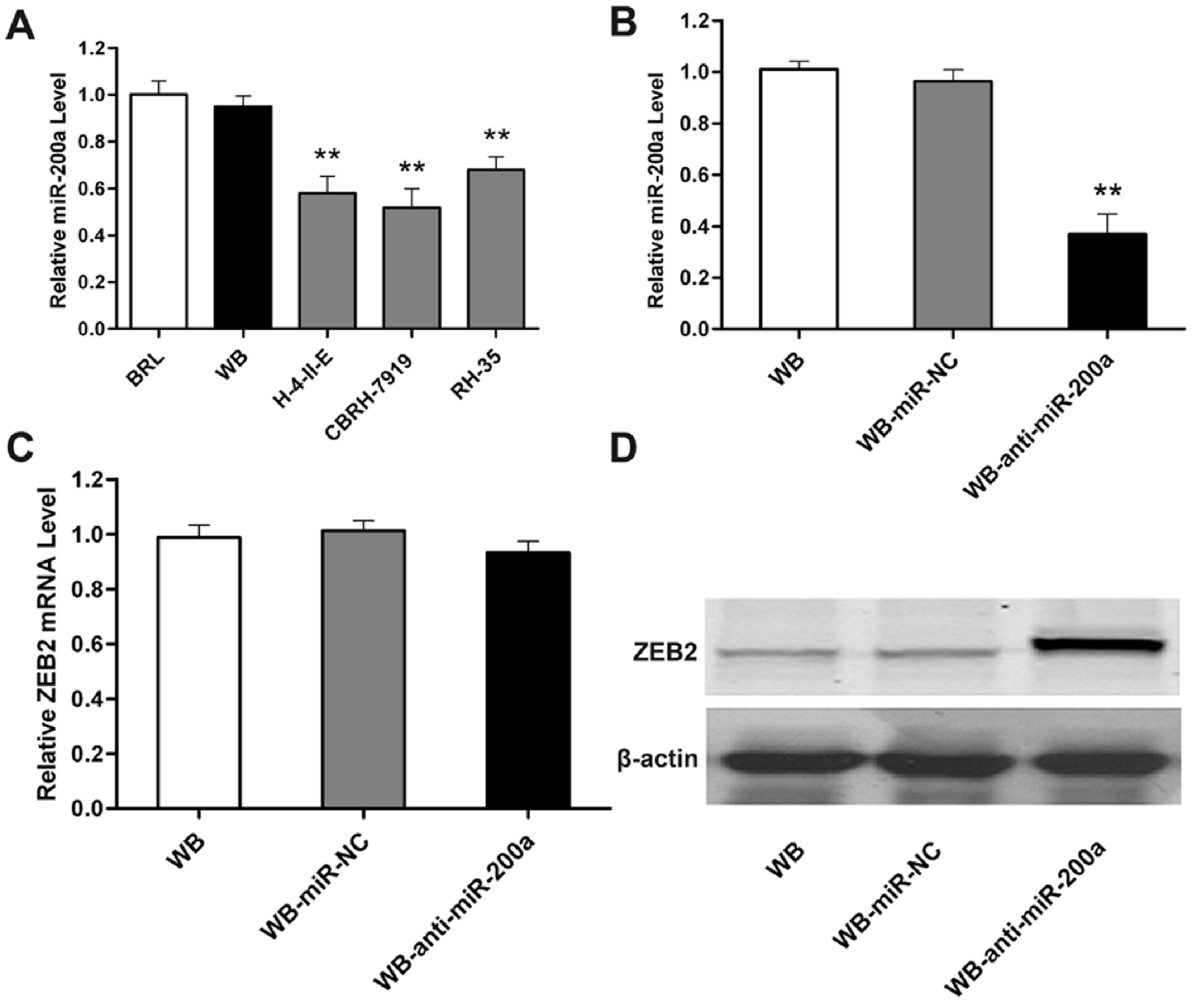
Expression levels of miR-200a and validation for stable miR-200a knockdown in WB cells. (A) QRT-PCR analysis of the relative miR-200a levels in WB cells and three hepatoma cells (H-4-II-E, CBRH-7919, RH-35) compared with the normal liver cell line BRL. (B) Validation of miR-200a levels in WB cells lentivirally transfected with miR-200a antagomir (WB-anti-miR-200a) or negative control (WB-miR-NC) by qRT-PCR analysis. (C and D) Functional evaluation of down-regulated miR-200a on its validated target ZEB2 in WB cells using qRT-PCR (C) and western blot analysis (D). For A and B, data are normalized to U6 and represented as the mean ± SD; n = 5; **, p<0.01. For C, data are normalized to β-actin and presented as the mean ± SD; n = 4.

To investigate the possible role of miR-200a on OCs, we first performed qRT-PCR to analyze the expression levels of miR-200a in WB cells as well as in BRL normal rat liver cells and three hepatoma cell lines (H-4-II-E, CBRH-7919, RH-35). Our results demonstrated that miR-200a expression was much higher in WB cells than in hepatoma cells (Figure 1A), indicating that down-regulated miR-200a may be associated with HCC malignant phenotypes. As a result, we introduced miR-200a loss-of-function studies in WB cells in the following research.

Next, we established lentivirus-mediated stable WB-anti-miR-200a and WB-miR-NC cell lines. QRT-PCR analysis showed the expression level of miR-200a in WB-anti-miR-200a cells decreased compared with WB-miR-NC cells, whereas its level was constant in WB-miR-NC cells versus WB cells (Figure 1B), suggesting that lentivirus infection was highly efficient and did not perturb endogenous miR-200a expression in WB cells. In addition, after miR-200a silencing in WB cells, mRNA expression of ZEB2, a well-acknowledged target of miR-200a, was constant (Figure 1C), whereas its protein expression increased (Figure 1D). These results indicate that miR-200a is effectively and functionally suppressed in WB-anti-miR-200a cells, serving as the basis of the remaining experiments.

### Stable Knockdown of miR-200a Facilitates CSC-like Phenotypes in WB Cells

**Figure 2:**
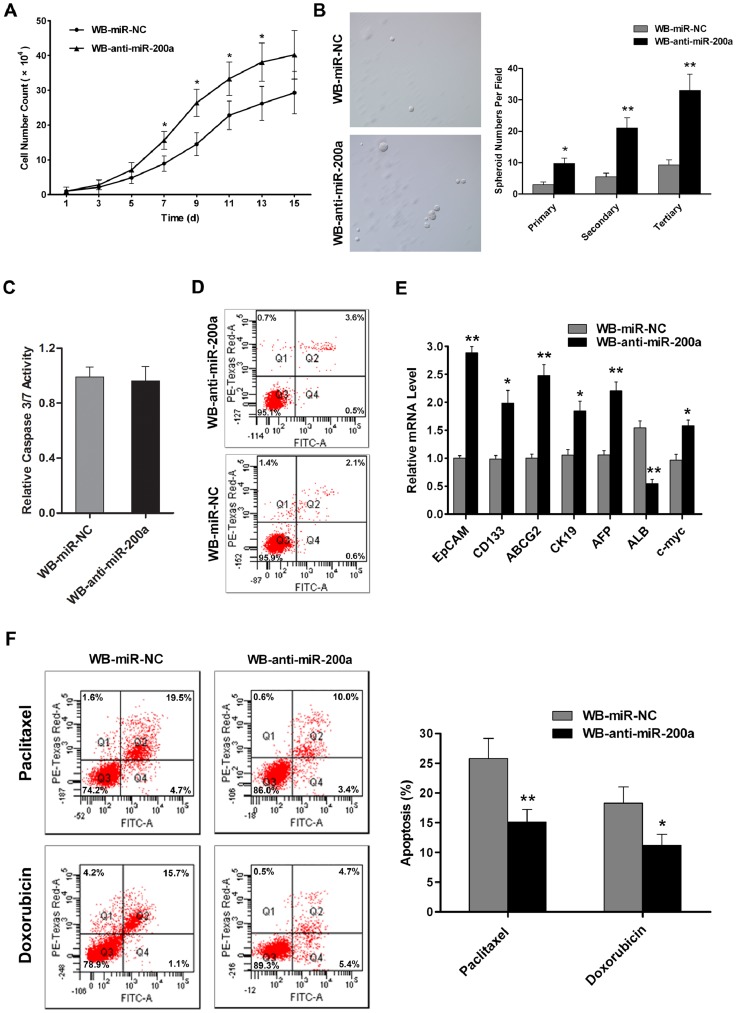
Stable knockdown of miR-200a facilitates CSC-like phenotypes in WB cells. (A) Growth curve of WB-miR-NC and WB-anti-miR-200a cells determined by cell counting. Data are expressed as the mean ± SD; n = 3; *, p<0.05. (B) Representative images of spheroids formed by WB-miR-NC and WB-anti-miR-200a cells in the spheroid formation assay (left, magnification×100). Number of spheroids formed in the primary, secondary and tertiary generations of suspension cultured WB-miR-NC or WB-anti-miR-200a cells (right). Data represent means from four randomly selected fields under the microscope, and error bars represent SD. *, p<0.05; **, p<0.01. (C and D) Apoptosis of WB-miR-NC and WB-anti-miR-200a cells measured by caspase-3/7 assay and Annexin V and PI staining. Data are expressed as the mean ± SD (C) and representative dot plots of apoptosis tests are shown (D); n = 5. (E) Expression of EpCAM, CD133, ABCG2, CK19, AFP, ALB and c-myc in WB-anti-miR-200a cells measured by qRT-PCR. Data are normalized to β-actin, shown relative to the level in WB-miR-NC cells and expressed as the mean ± SD; n = 3; *, p<0.05; **, p<0.01. (F) WB-miR-NC and WB-anti-miR-200a cells were treated with 10 ng/ml paclitaxel or 30 ng/ml doxorubicin for 48 h and then subjected to FACS with Annexin V and PI staining, respectively. Representative dot plots (left) and the mean percentage of apoptotic cells (± SD) from three independent experiments (right) are shown; *, p<0.05; **, p<0.01.

It has been demonstrated that CSCs with stem/progenitor cell characteristics possess excessive self-renewal capability [30]. In our study, cell proliferation assays showed that miR-200a silencing was associated with a time-dependent promotion of cell growth in WB cells (Figure 2A). Strikingly, in the spheroid formation assay, more spheroids were observed in suspension cultured WB-anti-miR-200a cells compared with WB-miR-NC cells, and the number of spheroids expanded even faster in WB-anti-miR-200a cells after serially passaging in both groups (Figure 2B), suggesting that miR-200a knockdown enhanced the self-renewal capacity of WB cells. In addition, the differences in cell proliferation and spheroid forming ability were not due to differences in apoptosis, because caspase-3/7 activity and the ratio of apoptotic cells were similar between miR-200a silencing cells and control cells (Figure 2C and 2D).

To further determine the characteristics of WB-anti-miR-200a cells, expression of putative hepatic CSC markers were examined by qRT-PCR. As shown in Figure 2E, expression of EpCAM, CD133, ABCG2, CK19 and AFP were much higher in WB-anti-miR-200a cells than in WB-miR-NC cells, whereas the ALB mRNA level was relatively downregulated, indicating that WB-anti-miR-200a cells are in a less differentiated state. Interestingly, expression of the oncogene c-myc was also up-regulated in WB-anti-miR-200a cells (Figure 2E), suggesting that miR-200a silencing may also promote WB cells to obtain transformed characteristics.

Chemo-resistance is one of the key hallmarks of CSCs. By assessing the apoptotic cells after exposure to anticancer drugs, we showed that WB-anti-miR-200a cells exhibited reduced apoptosis upon paclitaxel or doxorubicin (15.1±2.1% or 11.2±1.9%, respectively) treatment, in contrast to WB-miR-NC cells (25.8±3.4% or 18.3±2.7%, respectively) (Figure 2F), indicating that WB-anti-miR-200a cells might be more resistant to anticancer drugs. All together, these data strongly suggest that stable knockdown of miR-200a stimulates hepatic CSC-like characteristics in WB cells.

### Stable Knockdown of miR-200a Confers Mesenchymal Characteristics to WB Cells

**Figure 3:**
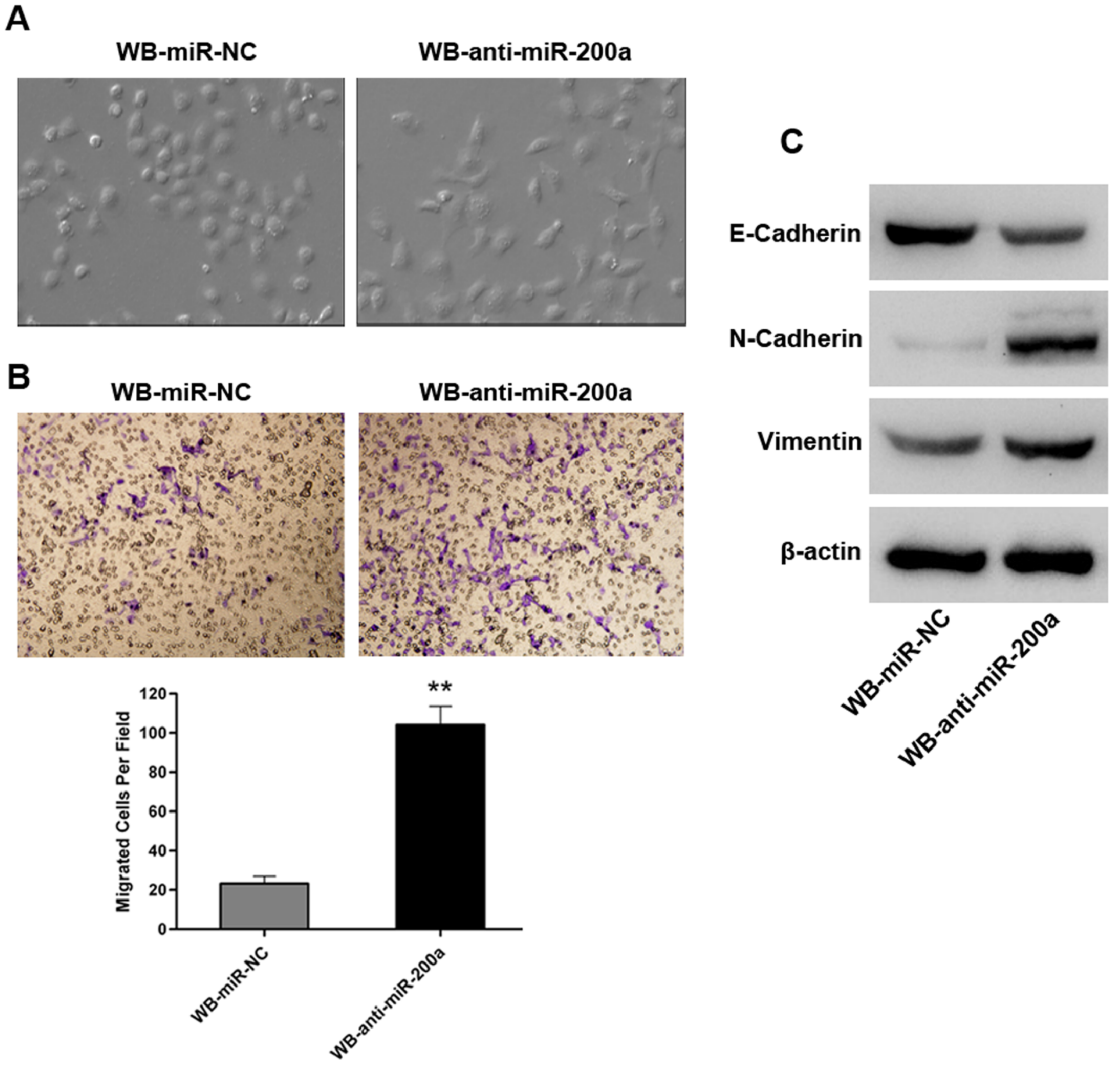
Stable knockdown of miR-200a confers mesenchymal characteristics to WB cells. (A) Morphological changes in WB-miR-NC and WB-anti-miR-200a cells (magnification×200). (B) Evaluation of in vitro migration abilities of WB-miR-NC and WB-anti-miR-200a cells by transwell migration assay. Representative images (upper, magnification×200) and the mean number of migrated cells (± SD) in five randomly selected fields counted under the microscope (lower) are shown; **, p<0.01. (C) Western blot analysis of epithelial (E-cadherin) and mesenchymal (N-cadherin and vimentin) markers in WB-miR-NC and WB-anti-miR-200a cells.

We obtained evidence that miR-200a knockdown might also be associated with an EMT-like phenotype in WB cells. Morphologically, WB-anti-miR-200a cells were partly spindle shaped similar to mesenchymal cells, whereas WB-miR-NC cells were tightly bound, oval-like cells with an epithelial phenotype (Figure 3A). Furthermore, in vitro migration assays revealed that WB-anti-miR-200a cells exhibited enhanced migration capacity compared with WB-miR-NC cells (Figure 3B). We also examined the protein expression of epithelial (E-cadherin) and mesenchymal (N-cadherin, Vimentin) markers by western blotting. As shown in Figure 3C, consistent with their elongated mesenchymal morphology, down-regulated E-cadherin and up-regulated N-cadherin, as well as Vimentin, were detected in WB-anti-miR-200a cells. Based on these morphologic changes as well as biological and biochemical behaviors shown in WB-anti-miR-200a cells, we conclude that miR-200a silencing induces the EMT-like phenotype in WB cells.

### Downregulated miR-200a could Directly Target CTNNB1 and Activate the Wnt/β-catenin Pathway in WB Cells

**Figure 4:**
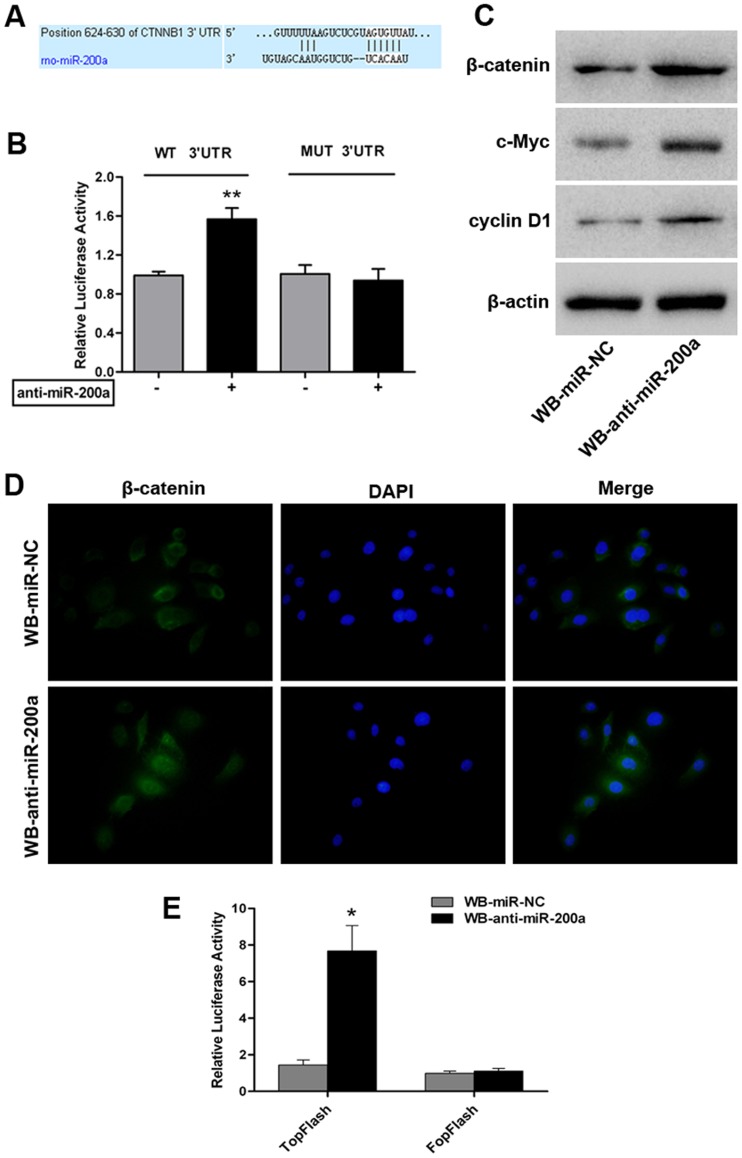
miR-200a directly targets CTNNB1 and associates with Wnt/β-catenin pathway activity in WB cells. (A) Predicted alignment of miR-200a and a potential binding site at the 3′-UTR of rat CTNNB1 mRNA (624–630 nt) by TargetScan. (B) Effect of miR-200a on CTNNB1 expression determined by a luciferase reporter assay. WB cells were co-transfected with anti-miR-200a (or anti-miR-control) and the pGL3-CTNNB1-wt (or pGL3-CTNNB1-mut) vector. Data are normalized by the ratio of Firefly and Renilla luciferase activities measured at 48 h post-transfection and are shown as the mean ± SD; n = 3; **, p<0.01. (C) Expression of β-catenin, c-myc and cyclin D1 in WB-miR-NC and WB-anti-miR-200a cells detected by western blot analysis. (D) Cellular localization of β-catenin visualized by immunofluorescence staining in WB-miR-NC and WB-anti-miR-200a cells. Nuclear counterstaining was performed by DAPI (magnification×200). (E) The activity of β-catenin signaling determined by a luciferase reporter assay using the wild-type (TopFlash) or mutant (FopFlash) TCF4-reporter plasmids. Data are presented as fold increases in firefly luciferase over Renilla activity from three independent experiments; *, p<0.05.

Accumulating evidence has demonstrated that the Wnt/β-catenin pathway plays an important role in oval cell activation, proliferation, and expansion in HCC subsets [31], [32], [33]. Thus, we postulated that miR-200a might target genes associated with Wnt/β-catenin signaling. By computational algorithms from TargetScan and mirBase target database, we found that the 3′-UTR of rat β-catenin (CTNNB1) mRNA contains a conserved putative miR-200a target site (Figure 4A). To validate the prediction, a 3′-UTR fragment of rat CTNNB1 mRNA containing wild-type or a mutated binding site for miR-200a was cloned into a luciferase reporter vector. The results showed that anti-miR-200a transfection significantly increased the expression of luciferase containing a CTNNB1-wt 3′-UTR binding site for miR-200a in WB cells compared with the control (Figure 4B). However, this stimulative effect was abrogated by mutations in the seed complementary sites of the 3′-UTR of CTNNB1 (Figure 4B). Moreover, we also performed western blot analysis to detect β-catenin protein levels in WB-anti-miR-200a cells and WB-miR-NC cells. As shown in Figure 4C, miR-200a silencing induced the protein expression of CTNNB1.

Accumulation of intracellular β-catenin, especially in the nucleus, is an indication of activated β-catenin-dependent signaling; hence, we further determined whether enhanced β-catenin expression impacted β-catenin pathway activity. The immunofluorescence staining data illustrated that β-catenin expression showed strong cytoplasmic and some nuclear localization when miR-200a was silenced in WB cells (Figure 4D). Additionally, WB-anti-miR-200a cells exhibited increased TopFlash luciferase activity compared with WB-miR-NC cells (Figure 4E). Moreover, the expression of cyclin D1 and c-myc, two representative target genes of the Wnt/β-catenin pathway, was also enhanced following the knockdown of miR-200a in WB cells (Figure 4C). Taken together, our data indicate that CTNNB1 mRNA is a direct target of miR-200a, and knockdown of miR-200a could activate, at least in part, the Wnt/β-catenin signaling pathway in WB cells.

### The Anti-miR-200a Effects can be Partially Attenuated by Silencing of CTNNB1 in WB-anti-miR-200a Cells

**Figure 5:**
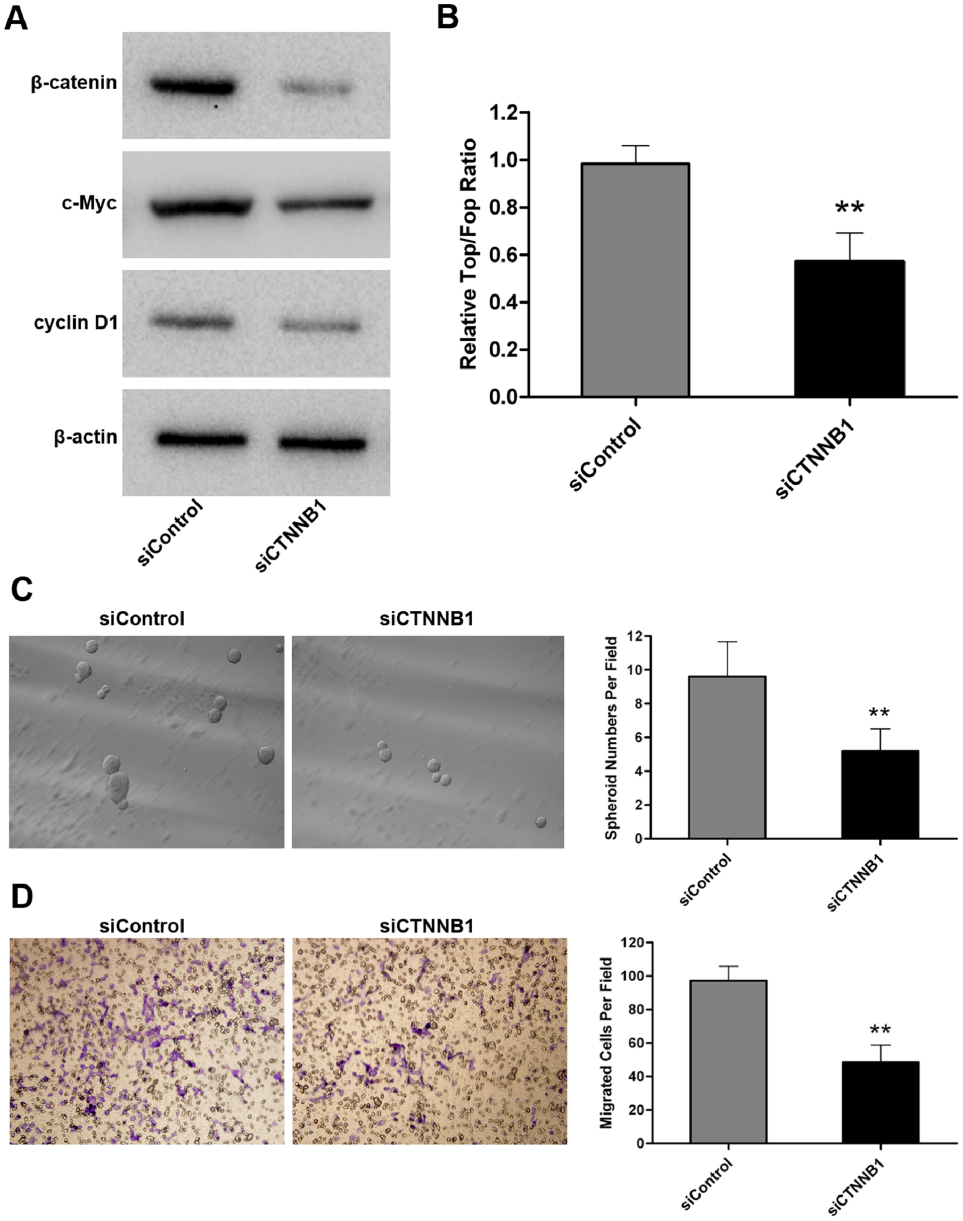
The anti-miR-200a effects are partially attenuated by silencing of CTNNB1 in WB-anti-miR-200a cells. (A) The protein levels of β-catenin, c-myc and cyclin D1 measured by western blot analysis after siCTNNB1 transfection in WB-anti-miR-200a cells. (B) The β-catenin-mediated transcription activity determined by the Top/Fop ratio after siCTNNB1 transfection in WB-anti-miR-200a cells. Data are represented as the mean ± SD; n = 3; **, p<0.01. (C) Representative images of spheroids formed by WB-anti-miR-200a cells before and after siCTNNB1 transfection (left, magnification ? 100). Bar graph (right) represents the mean number of spheroids from five randomly selected fields under the microscope, and error bars represent SD. **, p<0.01. (D) Representative images of transwell migration before and after siCTNNB1 transfection in WB-anti-miR-200a cells (left, magnification × 200). Bar graph (right) represents the mean number of migrated cells (± SD) in five randomly selected fields counted under the microscope. **, p<0.01.

To further elucidate whether elevated β-catenin expression and activated β-catenin pathway are functionally associated with miR-200a-silencing-mediated biological activities of WB cells, we used siCTNNB1 transfection and examined its effects on WB-anti-miR-200a cells. We confirmed that transfection of siCTNNB1 did not induce a detectable miR-200a expression change in WB-anti-miR-200a cells (data not shown). The silencing of CTNNB1 and inhibition of β-catenin-mediated transcription activity in WB-anti-miR-200a cells were confirmed by western blot analysis (Figure 5A) and Top/Fop luciferase reporter assays (Figure 5B), respectively. As expected, the stimulatory effect of miR-200a-silencing on cyclin D1 and c-myc expression was abolished by siCTNNB1 transfection (Figure 5A). Furthermore, we observed dramatic diminished spheroid formation (Figure 5C) and reduced migration abilities (Figure 5D) following CTNNB1 silencing in WB-anti-miR-200a cells. Collectively, these results demonstrate that activation of the Wnt/β-catenin pathway is functionally relevant to miR-200a-silencing-mediated biological activities of WB cells.

### Downregulation of miR-200a Confers Tumorigenicity to WB Cells in vivo

**Figure 6:**
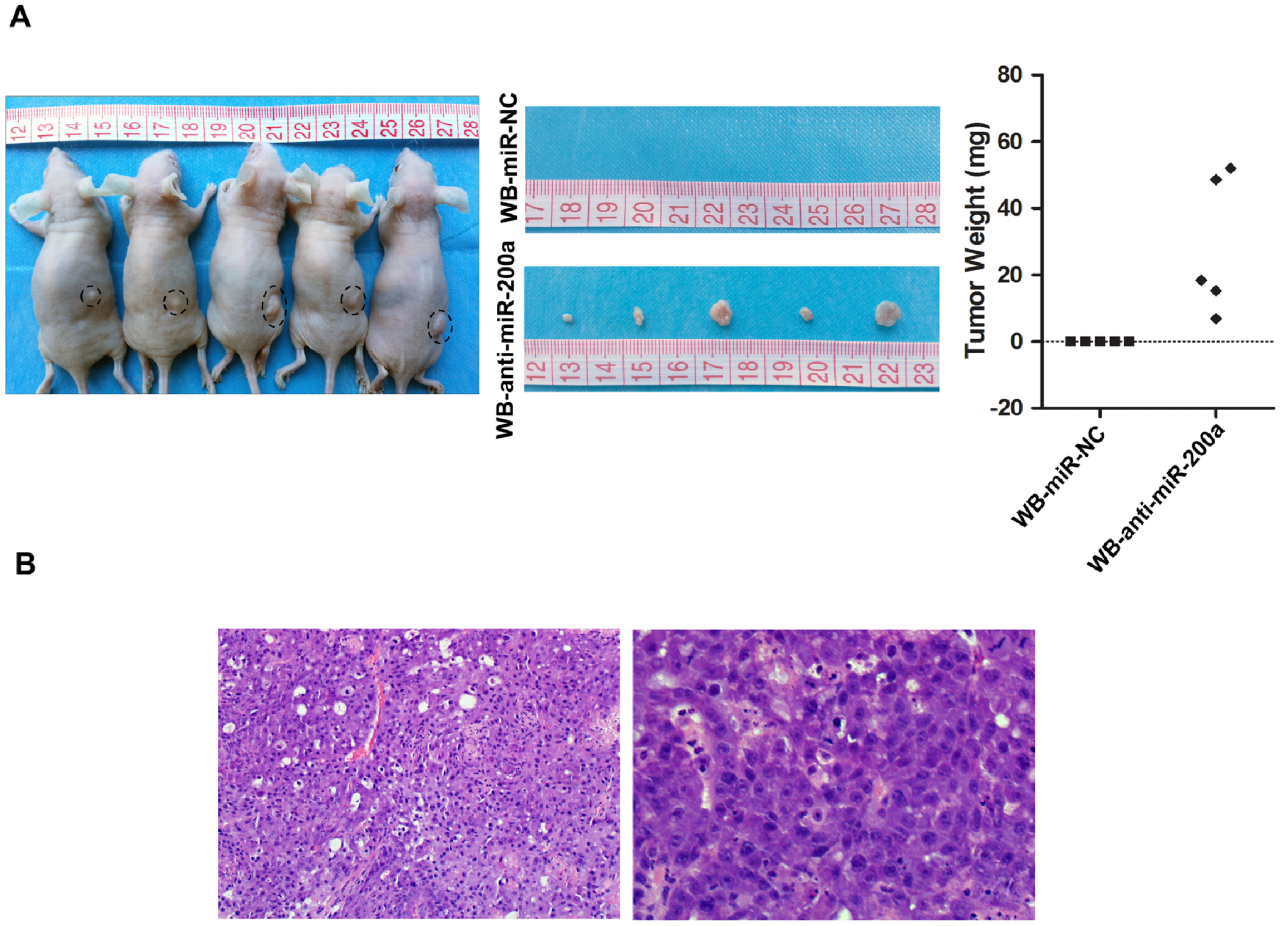
Downregulation of miR-200a confers tumorigenicity to WB cells in vivo. (A) Subcutaneous tumors developed by WB-miR-NC or WB-anti-miR-200a for 40 days post inoculation (left). Pictures of collected subcutaneous tumors were taken (middle) and tumor weights are shown (right). (B) Representative images of H&E staining of xenografted tumors are shown (left, magnification×200; right, magnification×400).

As in vitro studies showed that stable knockdown of miR-200a facilitates CSC-like signatures and EMT phenotypes in WB cells, we performed xenograft formation assay to further investigate the tumorigenicity mediated by miR-200a suppression. As shown in Figure 6A, WB-anti-miR-200a cells developed visible tumors on every right flank of the five nude mice, whereas no exhibited tumor was observed on the left flanks injected with WB-miR-NC cells. Histological analysis of subcutaneous tumors by H&E staining showed disorganized tumor cells, hyperchromatic nuclei and high nuclear-cytoplasmic ratio, which assembles HCC-specific features (Figure 6B). To conclude, the in vivo results indicated that downregulation of miR-200a in WB cells could induce tumorigenicity and finally give rise to tumors in nude mice.

## Discussion

With progress in the identification of CSCs, emerging evidence strongly support the notion that HCC is driven by a small population of cells through their excessive self-renewal capacity, production of heterogeneous progeny, resistance to chemotherapy and limitless dividing [34]. Recently, recognition of the role of HOCs in the carcinogenic process prompted a new belief that HCC might arise by maturation arrest of liver adult stem cells [35]. In other words, hepatic CSCs could originate from normal HOCs. As an important regulatory factor, the miR-200 family has been identified as suppressors of CSC-like or EMT-like phenotypes and functions in various normal and cancer cells [22], [23], [24], [36]. Our previous study also showed that miR-200a was greatly downregulated in the F344 rat HCC SP fraction cells compared with their normal counterparts [26]. In this study, using loss-of-function studies, we further investigated the function of miR-200a on HOCs.

The WB-F344 epithelial cell line was first derived from the liver of an adult male Fischer 344 rat [27] and has been proposed to be an in vitro model of HOC [28]. As an initial step, the expression pattern of miR-200a was assessed in the WB-F344 cell line, BRL normal liver cell line and three other hepatoma cell lines (H-4-II-E, CBRH-7919, RH-35). We provide evidence that miR-200a expression was relatively higher in WB cells and BRL cells than in three other rat hepatoma cell lines. These results were also in line with previous studies reporting downregulation of miR-200a in poorly differentiated HCC cell lines compared with well differentiated cell lines and in HCC tissues compared with the adjacent noncancerous hepatic tissues [37], [38]. Therefore, it can be calculated that dysregulated miR-200a may be intimately associated with malignant phenotypes of HCC.

Next, using a lentiviral vectors approach, we transfected WB cells with miR-200a antagomirs and stably silenced miR-200a in WB cells, results that were confirmed by qRT-PCR and western blotting. Functional studies showed that WB cells stably lacking miR-200a exhibited CSC-like properties. Four lines of evidence support this finding. First, stable knockdown of miR-200a enhanced proliferation and conferred a greater potential for self renewal capacity even after serial passages in WB cells. Second, putative hepatic CSC markers (EpCAM [30], CD133 [8] and ABCG2 [39]) were up-regulated after miR-200a knockdown. Third, WB-anti-miR-200a cells were in a less differentiated state by higher expression of primitive liver markers AFP and CK19, and lower expression of the mature liver marker ALB. Fourth, miR-200a silencing led to superior anti-apoptosis capacity to chemotherapeutic drugs in WB cells. Specifically, although more evidence is needed, it can be postulated that this chemo-resistant phenomenon might be due to the above observed increased expression of ABCG2, which is a universal CSC marker always implicated in resistance to chemotherapeutic agents [40], [41].

EMT is a critical process involved in cancer progression and metastasis. During EMT, loss of epithelial markers and gain in the expression of mesenchymal markers facilitate cells to undergo changes in cell morphology, which is accompanied by enhanced cell motility and migration [42]. In addition, emerging data also suggest a role for this process in generating CSC-like features in epithelial cells [43], [44], [45]. Recently, it has been confirmed that the miR-200 family plays a key role in negatively regulating the EMT process [21]. More strikingly, miR-200 family members have been reported to be directly regulated by p53, further highlighting their role in tumor progression [46]. In our study, WB cells stably lacking miR-200a also presented mesenchymal characteristics, including elongated cell morphology, enhanced cell migration ability, down-regulated E-cadherin and up-regulated N-cadherin, as well as Vimentin. This is the first report, to the best of our knowledge, demonstrating that downregulation of miR-200a is associated with EMT and CSC-like phenotypes in HOCs. Our finding is in accordance with phenomena previously observed in pancreatic cells [47] and nasopharyngeal carcinoma cells [48], indicating that miR-200a indeed plays an extensive and profound role in the context of cancer initiation and progression.

Regarding the molecular mechanisms underlying the role of miR-200a in WB cells, we used combined bioinformatics and experimental approaches to identify CTNNB1 as a direct functional downstream target of miR-200a. CTNNB1 encodes β-catenin, which is not only an essential component of the cadherin adhesion protein complex to regulate epithelial cell integrity and polarity but also a central modulator of the canonical Wnt signaling pathway. It has been well established that accumulated cytosolic β-catenin can translocate into the nucleus and then form the β-catenin/TCF complex to activate transcription of Wnt target genes [49]. Consistent with these theories, we demonstrated in this study that stable knockdown of miR-200a enhanced the β-catenin protein level. Moreover, elevated β-catenin showed strong cytoplasmic and nuclear localization, which is consistent with increased TopFlash luciferase activity and enhanced expression of Wnt target genes, c-myc and cyclin D1. In addition, a previous study found that EpCAM can act as a Wnt downstream molecule to maintain stemness properties in HCC subsets [30]. Thus, it is reasonable to speculate that activated β-catenin in this study might also account for the elevated EpCAM expression that was shown previously.

The Wnt/β-catenin pathway is a highly conserved signaling cascade participating in various cellular processes, including cell proliferation, differentiation, migration, adhesion, and survival [50]. Recently, Wnt/β-catenin signaling has been reported to correlate with an EMT-like states [51] and CSC genesis [52], self-renewal [53] and chemo-resistance [54] in many cancers. More importantly, the literature has shown that activation of Wnt/β-catenin signaling confers HOCs/HPCs with high self-renewal potential and tumorigenesis capacity in the liver [12], [32]. Consistent with their findings, a rescue experiment in our study found WB-anti-miR-200a transduced with siCTNNB1, but not scrambled siControl, showing diminished spheroid formation and reduced migration ability in vitro. The function of miR-200a exerting on WB cells is indeed, at least in part, attributed to β-catenin regulation in Wnt/β-catenin signaling.

It has been discovered that one miRNA can possibly target multiple genes in regulating cellular processes. In previous studies, miR-200a regulating the EMT process by targeting EMT-activating transcriptional factors ZEB1 and ZEB2 has been thoroughly elaborated in different types of cancers [55], [56]. In our study, we also found knockdown of miR-200a led to overexpression of ZEB2 protein in WB cells. Thus, in addition to directly targeting CTNNB1 and regulating the Wnt/β-catenin pathway, there is a possibility that miR-200a may also exert its function on WB cells via targeting ZEB2. Thus, more in-depth investigations are required to determine the complex interactions of miR-200a-dependent molecular regulatory network systems in HOCs.

In addition to in vitro findings, the xenograft tumorigenicity assay further confirmed the contributions of miR-200a silencing to malignant transformation of WB cells in vivo. Acquisition of tumorigenicity in oval cell lines is not unusual since it has been reported that long time exposure of WB-F344 cells to TGF-β or transfection with HBx gene in LE/6 cells (another HOC line) following treatment with aflatoxin B_1_ obtained same results [9], [57]. Moreover, except for EMT process and β-catenin pathway, miR-200a has also been found participating in epigenetic modulation through histone deacetylase 4/SP1/miR-200a regulatory network [38]. Thus, it can be calculated that miR-200a may have significant influence on cell functions through extensive mechanisms which needs further exploring.

To conclude, our data suggested that downregulation of miR-200a in liver oval cells promotes EMT and CSC-like traits in vitro, partially through the regulation of β-catenin signaling, and finally give rise to tumorigenicity in vivo. These findings underscore the importance of miR-200a in inhibiting neoplastic phenotypes in normal adult hepatic progenitor cells. Exploring miR-200a-based prevention and therapeutics might benefit HCC clinically.
